# A1- and A2 beta-casein on health-related outcomes: a scoping review of animal studies

**DOI:** 10.1007/s00394-021-02551-x

**Published:** 2021-06-01

**Authors:** Daniela Kuellenberg de Gaudry, Szimonetta Lohner, Karin Bischoff, Christine Schmucker, Simone Hoerrlein, Christine Roeger, Lukas Schwingshackl, Joerg J. Meerpohl

**Affiliations:** 1 Institute for Evidence in Medicine, Medical Center - University of Freiburg, Faculty of Medicine, University of Freiburg, Breisacher Str. 153, 79110 Freiburg, Germany; 2 Cochrane Hungary, Clinical Center, Medical School, University of Pécs, Pecs, Hungary; 3 Competence Center for Nutrition, Bavarian State Ministry for Food, Agriculture and Forestry, Freising, Germany

**Keywords:** A1 beta-casein, A1-milk, A2-milk, Beta-casein, Animal studies

## Abstract

**Purpose:**

Food-derived bioactive peptides may influence important physiological functions. An important example is beta-casomorphins, which are opioid peptides derived from A1 beta-casein in bovine milk and have been associated to be risk factors for non-communicable diseases in humans. A1 and A2 beta-casein are different with respect to the release of bioactive peptides, in particular BCM-7. However, evidence from human studies is limited and could be complemented with evidence derived from animal studies. We conducted a scoping review to identify animal studies investigating the effects of A1 beta-casein or BCM-7 compared to A2 beta-casein or any other intervention on health-related outcomes.

**Methods:**

We systematically searched for relevant studies in two electronic databases (Medline, Embase; last search performed March 2020). Two reviewers independently undertook study selection and data extraction of included references. Results were summarized tabularly and narratively.

**Results:**

We included 42 studies investigating various animal models, including rats, mice, rabbits, and dogs. Six studies investigated health-related outcomes of A1- vs. A2 milk, while most studies (*n* = 36) reported on physiological properties (e.g., analgesic effect) of BCM-7 as an opioid peptide. Included studies were extremely heterogeneous in terms of the study population, type of intervention and dose, and type of outcome measures.

**Conclusions:**

Only a few studies comparing the effects of A1- and A2 milk were identified. More studies addressing this research question in animal models are needed to provide essential information to inform research gaps. Results from future studies could eventually complement research for humans, particularly when the body of evidence remains uncertain as is the case in the A1- and A2 milk debate.

**Supplementary Information:**

The online version contains supplementary material available at 10.1007/s00394-021-02551-x.

## Background

Proteins are a broad family of organic compounds with an important role in the structure and functionality of all living organisms [1]. They are a fundamental component of animal and human diets providing a source of energy, nitrogen, and essential amino acids [1]. In addition, dietary proteins may also provide a source of biologically active peptides, which are inactive within the sequence of the precursor protein but may become active when released by hydrolysis during food processing [1]. Food-derived bioactive peptides may influence physiological functions, including modulation of gut secretion and motility, blood pressure, thrombotic, antioxidant, antimicrobial, and immunomodulatory activities [1]. Some of these effects are mediated by interaction with the opioid system and are therefore called opioid peptides.

Opioid peptides can be formed from milk, cereal, vegetables, and meat/poultry. The most investigated so far are the ones derived from bovine milk [1]. Approximately one-third of bovine milk proteins are beta-caseins, which are present in various genetically determined forms in milk, including the A1 and A2 variants. Milk free of A1 beta-casein is commonly known as A2 milk, which contains mostly A2 beta-casein with a possible caveat of minor contamination. All other bovine milk is commonly referred as to A1 milk, which contains predominantly A1 beta-casein. Both milk types have shown to have different digestive results. In contrast to the A2 milk, digestion of A1 milk releases beta-casomorphin 7 (BCM-7), which is a potent opioid peptide influencing the above-mentioned physiological effects [2]. Thus, BCM-7 is thought to be responsible for potential adverse outcomes in humans, such as the increased risk of diabetes [3]. In contrast, the consumption of milk containing exclusively the A2 beta-casein variant (A2 milk) has been promoted as being associated with positive health effects in humans, including reduced gastrointestinal symptoms [4].

We recently published a systematic review about the health effects of A1 milk reported in human studies and found some evidence suggesting beneficial gastrointestinal effects of A2 milk compared to A1 milk [5]. However, implications of A1 beta-casein on other health-related outcomes were limited and were graded with low or very low certainty of evidence. In contrast, various animal studies suggest that opioid peptides like BCM-7 could have even beneficial physiological properties [2].

The main objective of this scoping review was to identify and describe all primary studies evaluating health-related outcomes of A1 beta-casein consumption/exposure in animal models.

## Materials and methods

This scoping review was conducted following the methods from the Cochrane Handbook for systematic reviews of interventions [6], and following recommendations from the SYRCLE (Systematic Review center for Laboratory Animal Experimentation) method group [7]. Methods for the scoping review were defined a priori in a protocol (not registered). The methodology and the results are reported according to the PRISMA guidelines for scoping reviews [8] (see additional file 1).

### Eligibility criteria

The research question was defined according to the PICO approach (Participants, Interventions, Comparison, Outcomes). We included studies investigating A1 beta-casein or BCM-7 intake (independently of the method of administration, e.g., orally or given through injection) on any health-related outcome in animals (e.g., gastrointestinal markers such as gastrointestinal transit time (GITT), incidence of diabetes, intermediate disease markers of CVD such as LDL and HDL concentrations, etc.). Any comparison intervention (including A2 beta-casein, other BCM-fractions, etc.) was considered eligible, and included studies had to report on a health-related outcome. No restrictions on study design were applied. Studies published in a language other than English, Spanish, German, or French; or studies without a full-text (i.e., abstracts) were excluded, but listed in a separate table. Studies in which the intervention was given to deceased animals were excluded.

### Systematic literature search

To identify all published studies investigating A1 beta-casein on health-related outcomes in animals, we searched Medline (PubMed) and Embase from inception until February 2017. An update search was performed in March 2020. The search strategy was constructed using free text and MeSH terms (or EMTREE). To identify animal studies, the recommended filters from SYRCLE for both databases were used [9, 10]. Search strategies are presented in the supporting information (see additional file 2). Additionally, reference lists of eligible articles were screened for further relevant references.

### Study selection

Each reference was screened by two reviewers independently from each other (SL, DK, and KB) based on predefined inclusion criteria using the Covidence online software [11] in dual screening mode, i.e., each reference had to be screened by two of the three reviewers—the assignment of references is aleatory. First, titles and abstracts of studies retrieved through the searches were screened to exclude obvious irrelevant references. Second, full publications of potentially relevant studies were obtained and checked for final inclusion. Any disagreement was resolved through consensus.

### Data extraction

Data from each included reference were extracted by two reviewers independently from each other (DK, SL and KB—references were assigned aleatory to each reviewer by reference ID number), and any disagreement was resolved through consensus.

The following data were extracted for each included study: bibliographic details, study characteristics [incl. objective(s), details of funding, study design, number of animals included], characteristics of the included animals (incl. age, gender, animal species, breeding, housekeeping- and acclimatisation conditions), characteristics of the intervention and control intervention (incl. type, dosage, and mode of administration), and outcome data (incl. definition, time of measurement).

### Data synthesis

Results from this scoping review were summarized in bubble charts and in tables with relevant information on each included study. Results were also described narratively.

## Results

### Results of the search

**Fig. 1:**
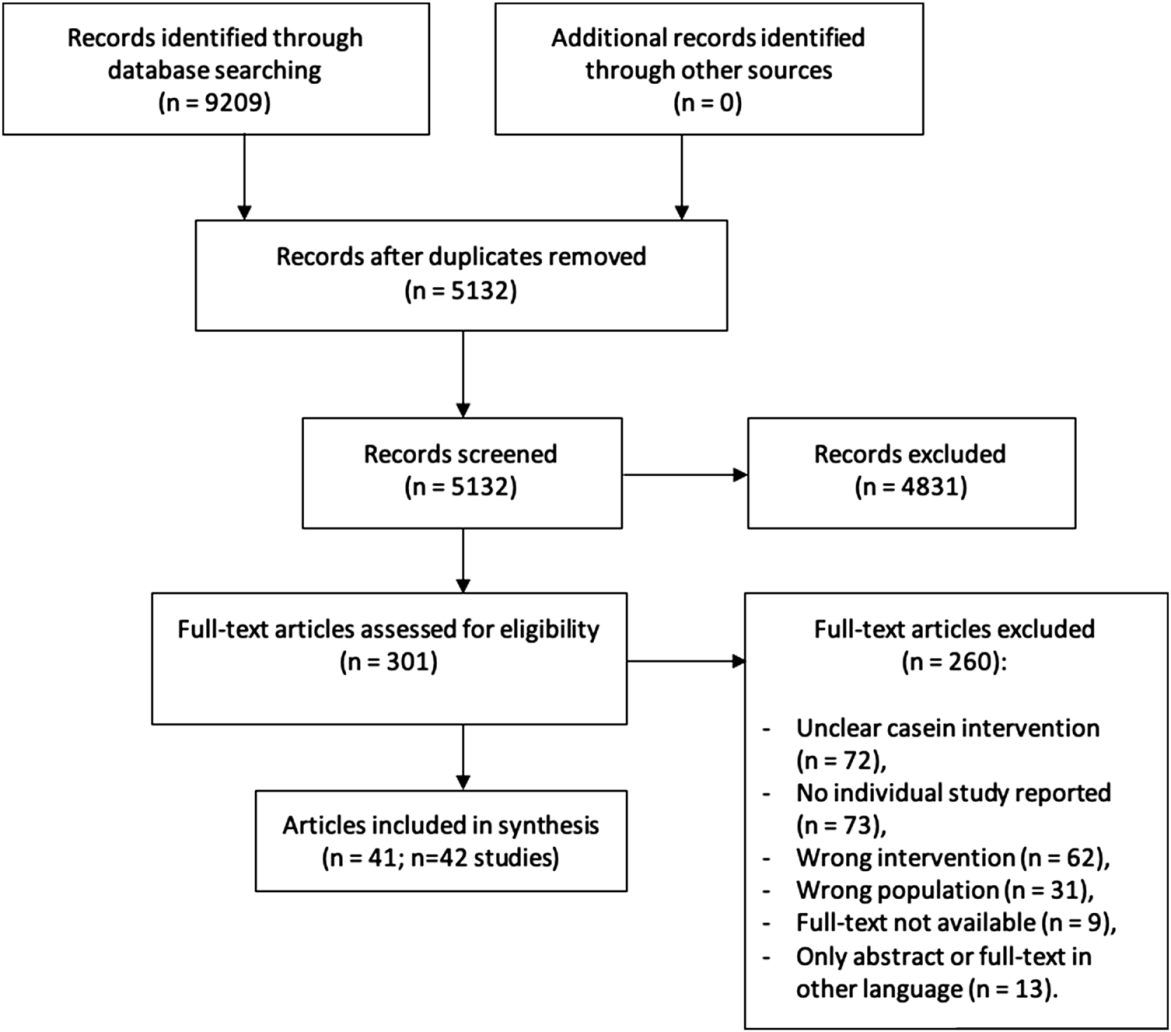
PRISMA flow diagram of systematic literature search

During the systematic literature search in both electronic databases, we identified 9209 potentially relevant records. After removing duplicates, 5132 unique records were assessed for eligibility. From these, 4831 records were excluded after title- and abstract screening and 260 after full-text screening. Finally, 41 records fulfilled our inclusion criteria (Fig. 1). From these, one reference reported results of two studies, therefore, 42 studies were included in this scoping review. Additionally, 14 records were excluded from data extraction, because they were published in other language, were not published as full texts, or full texts were not available (these references are listed in additional file 3).

### Description of included studies

A total of 42 trials were included in this scoping review. Six from the included studies evaluated A1- vs. A2 beta-casein, and the remaining 36 studies analysed BCM-7 vs. various control interventions, incl. other protein fragments (mainly shorter BCM fragments), distilled water or saline solution. Results of this scoping review are displayed for each of these two comparisons separately and can be found in Tables 1, 2 respectively.

**Table 1:** Characteristics of included studies and reported outcomes (comparison A2- vs. A2 beta-casein)

	Study	Country	Study population	Aims of the study (short description)	Intervention (I) / Control intervention (C)	Dose	Summary on reported outcomes	Study funding
1	Barnett 2014 [16]	New Zealand	48 male Wistar rats, 4 weeks old	To measure gastrointestinal effects of A1 or A2 beta-casein on rats fed for 36 or 84 h	I: Skim-milk diets containing A1 beta-casein C: Skim-milk diets containing A2 beta-casein	Not reported	Gastrointestinal markers* (incl. gastrointestinal transit time (GITT), intestinal inflammation)	A2 Corporation Limited and the New Zealand Government Foundation for Research Science and Technology (FRST)
2	Beales 2002 [12]	New Zealand, Canada and UK	315 non-obese diabetic (NOD) mice, aged 17–21 days; and 270 BioBreeding (BB) rats, aged 23 days	To ascertain whether A1 beta-casein was more diabetogenic than A2 and to test the diabetogenicity of a milk-free diet in animals representing different forms of spontaneous Type I (insulin-dependent) diabetes mellitus	I: Oral diets containing A1 beta-casein: (a) Hydrolysed casein based formula (Progestimil) with A1 beta-casein, or (b) Soy isolate based infant formula (ProSobee) with A1 beta-casein C: Various oral diets: (a) Progestimil, (b) ProSobee, (c) Progestimil containing whole casein, (d) Progestimil with A2 beta-casein, (e) ProSobee with A2 beta-casein, or (f) plant-based diet without milk proteins and containing mainly wheat (NTP-2000)	Not reported	Intermediate markers related to diabetes*: glucose concentration in blood and urine Insulitis (inflammation of the islets of Langerhans of the pancreas) Survival Body weight	New Zealand Dairy Board, Juvenile Diabetes Research Foundation, Canadian Institutes of Health Research, und Ontario Research and Development Challenge Fund and Health Canada
3	Chia 2018 [13]	Australia	Newly weaned NOD/ShiLtJArc mice, aged 3–4 weeks; and their breeded further 4 generations of mice (total number of included animals was not reported)	To test whether a diet supplemented with A1 or A2 beta-casein would increase the incidence of type 1 diabetes in genetically susceptible female NOD mice over generations	I: Oral administration of A1 beta-casein supplement with normal diet C: Oral administration of A2 beta-casein with normal diet	Not reported	Outcomes were reported for the included mice and their further 4 generations of breeded mice: Incidence of diabetes* Blood parameters, incl. glucose, insulin, immune profile, etc Gut microbiota, and permeability	Innovation Connections Grant (Nr. RC54051) of the Department of Industry, Innovation and Science, Australia; and a2 Infant Nutrition Australia Private Limited, Sydney, Australia
4	Kaminski 2012 [15]	Poland	6 pigs (cross of Polish Large White and Polish Landrace), 83 days old and weighing 33 kg	To verify the hypothesis whether consumption of cow's milk containing A1 variant or A2 variant of beta-casein will affect basic parameters of blood	I: Oral administration of A1 milk (as supplement) C: Oral administration of A2 milk (as supplement)	Dose increased during the study and each pig received the following dose (either I or C): Week 1: 0 kg/day Week 2: 0.32 kg/day Week 3: 0.54 kg/day Week 4: 0.72 kg/day Week 5: 1 kg/day Week 6: 1.5 kg/day	Intermediate markers related to CVD*: blood lipids Intermediate markers related to diabetes*: glucose concentration Other blood parameters (incl. white blood cells, red blood cells, platelets, creatinine, urea)	University of Warmia and Mazury (No. 0105-0804)
5	Tailford 2003 [17]	Australia	60 rabbits (New Zealand white/Lop cross rabbits), aged 16–24 weeks	To determine whether dietary administration of beta-casein A1 in a rabbit model of atherosclerosis promotes the disease state compared with rabbits fed beta-casein A2	I: Various intervention groups with different concentrations of A1 beta-casein given orally (pellets) C: Various control groups with different concentrations of A2 beta-casein and with or without whey protein given orally (pellets)	Oral diets had the following concentrations of either A1- or A2 beta-casein: 10%, 3.5%, or 20% Rabbits were fed with one of the diets for 6 weeks and the number of pellets eaten by each rabbit was recorded daily	Intermediate marker related to CVD (atherosclerosis)*: aortic fatty streak and advanced lesions in carotid arteries Body weight Various blood parameters (incl. blood lipids, homocysteine)	Not reported
6	Haq 2014a [14]	India	24 Swiss albino male mice, weighing between 20 and 25 kg	To study the effect of feeding three genetic variants (A1A1, A1A2, and A2A2) of cow beta-casein milk on gastrointestinal immune system of mice	I: Oral administration (intubation) of: (a) A1 beta-casein, or (b) A1- and A2 beta-casein C: Oral administration (intubation) of A2 beta-casein	Mice received the following dose of either A1- or A2 beta casein: 85 mg/mice/day for 30 days	Gut immune response*, measured with immunoglobulins, intestinal leucocyte infiltration, etc	National Dairy Research Institute (ICAR)

**Table 2:** Characteristics of included studies and reported outcomes (comparison BCM-7 vs. any other intervention)

	Study	Country	Study population	Aims of the study (short description)	Intervention (I)/Control intervention (C)	Dose	Reported Outcomes	Study funding
1	Blass 1996 [23]	USA	120 Sprague–Dawley rats, 10 days old	To evaluate effects of i.p. injections of BCM4, BCM5 and BCM7 on thermal pain in 10-day-old rats	I: BCM-7 administration (i.p.) C: Administration (i.p.) of either: (a) BCM-4, (b) BCM-5, or (c) isotonic saline solution	Single dose of either I or C with the following concentrations: 0.1 mg/kg, 0.5 mg/kg, 1 mg/kg, 2.5 mg/kg	Neurological effect*: analgesic effect	Not reported
2	Brantl 1981 [18]	Germany	Sprague–Dawley rats (approx. 8 rats, no further details reported)	To test the opioid activity of BCM7	I: BCM-7 administration (i.c.v.) C: Administration (i.c.v.) of either: (a) BCM-3, (b) BCM-4, (c) BCM-5, or (d) BCM-6	Single dose of 10 μl of BCM dissolved in saline solution (corresponds to approx. 0.06–2 μmol BCM)	Neurological effect*: analgesic effect	Not reported
3	Chang 2019 [24]	China	120 male Arbor Acres broiler chickens, 21 days old, with similar body weight	To investigate effect of BCM on lipid metabolism in broiler chickens and its mechanism	I: BCM-7 administration (subcutaneously) C: saline solution	I: Daily either 0.1 mg/kg, 0.5 mg/kg, or 1 mg/kg in 1 ml saline solution for 7 days C: 1 ml	Fat deposition* Growth performance Differential expression of genes in the liver Blood parameters (blood lipids and hormone levels)	China agricultural research system (CARS-41-G08), and the Agricultural Science and Technology Innovation Program (ASTIP)
4	Dubynin 1992 [29]	Russia	104 noninbred male albino rats, weighing between 150 and 250 g	To study dependence of the analgesic activity of BCM7 on dose and on the initial pain sensitivity of experimental animals	I: BCM-7 administration (i.p.) C: Administration (i.p.) of distilled water	Single dose of either I or C in the following concentrations: 5 mg/kg, 10 mg/kg, or 20 mg/kg	Neurological effect*: analgesic effect	Not reported
5	Dubynin 1998 [30]	Russia	365 male albino rats, weighing 200 g	To evaluate the effects of wheat gluten fragments, hemoglobin, and milk B-caseins (exorphine C, hemorphine-6, and BCM7) on nociceptive sensitivity and behaviour	I: BCM-7 administration (i.p.) C: Administration (i.p.) of either: (a) hemorphine-6, (b) exorphine-C, or (c) distilled water	Single-dose of either I or C with the following concentrations: 5 mg/kg, or 20 mg/kg	Neurological effect*: analgesic effect	Russian Foundation for Basic Research (Nr. 97-04-48166)
6	Dubynin 2000 [31]	Russia	98 outbred albino rats, aged 10–23 days	To evaluate delayed behavioural effects of BCM7 administered during the early ontogeny	I: BCM-7 administration (i.p.) C: Administration (i.p.) of distilled water	Daily 1 mg/kg of either I or C (duration of the intervention was not reported)	Neurological effect*: analgesic effect	Russian Foundation for Basic Research (Nr. 99-04-48410)
7	Dubynin 2007 [32]	Russia	480 young outbred white rats, aged 10 and 17 days	To evaluate the effect of BCMs on mother-oriented behaviour of white rats	I: BCM-7 administration (i.p.) C: Administration (i.p.) of three shorter fragments of BCM or naloxone (no further details reported)	Single-dose of 5 mg/kg of either I or C	Neurological effect*: behavioural change (mother-oriented behaviour)	Russian Foundation for Basic Research (Nr. 05-04-49761) und the Basic Research Program of the Presidium of the Russian Academy of Sciences “Molecular and Cell Biology”
8	Dubynin 2008 [33]	Russia	85 juvenile outbred albino rats (males and females)	To study the effect of chronic administration of BCM7 on the learning of albino rat pups	I: BCM-7 administration (i.p.) C: equal solvent volumes (no further details reported)	Daily 1 mg/kg of either I or C for 14 days	Neurological effect*: behavioural change (learning behaviour)	Russian Foundation for Basic Research (Nr. 05-04-49761) und the Basic Research Program of the Presidium of the Russian Academy of Sciences “Molecular and Cell Biology”
9	Gritsai 2000 [34]	Russia	300 laboratory cultured cockroaches (Periplaneta Americana), weighing approx. 1 g	To evaluate the locomotor defence response (LDR) to electrical stimulation after injection of BCM7	I: BCM-7 administration (between the bases of the forelegs) C: Administration (between the bases of the forelegs) of: (a) naloxone, or (b)saline solution	I: single dose of either 50 μg/g, 100 μg/g, or 200 μg/g C (a): either 50 μg/g, 5 μg/g, or 1 μg/g C (b): dose not reported	Neurological effect*: analgesic effect	Not reported
10	Han 2013 [43]	China	32 Sprague–Dawley rats, weighing between 150 and 180 g, aged 32 weeks	To evaluate efficacy of BCM7 against myocardial injury in streptozotocin- induced diabetic rats	I: Oral administration of BCM-7 with the normal diet C: normal diet	I: daily 7.5 × 10^–8^ mol for 30 days C: n.a	Intermediate marker related to diabetes*: cardiac index (diabetic cardiomyopathy) Various enzyme concentrations Blood glucose concentration Body weight change	National Natural Science Foundation of China und Priority Academic Program Development of Jiangsu Higher Education Institutions (PAPD)
11	Haq 2014b [49]	India	18 Swiss albino mice, weighing between 20 and 25 kg	To evaluate the influence of BCM5/7 on the inflammatory immune response in the mice gut	I: Oral administration (intubation) of BCM-7 C: Oral administration (intubation) of either: (a) BCM-5, or (b) PBS	I: daily 7.5 × 10^–8^ mol dissolved in 200 μl PBS for 15 days C (a): daily 7.5 × 10^–8^ mol dissolved in 200 μl PBS for 15 days C (b): daily 200 μl PBS for 15 days	Gut immune response*, measured with immunoglobulins, the concentration of various enzymes, etc.	National Dairy Research Institute (ICAR)
12	Hedner 1987 [19]	Sweden	Danish rural breed rabbits, weighing approx. 45 g (no further details reported); and approx. 34 male Sprague–Dawley rats, weighing between 225 and 325 g	To evaluate and compare ventilatory effects of BCM7, BCM5, BCM4 and morphine	I: BCM-7 administration (i.v, i.p., or i.c.v.) C: Administration (i.v, i.p., or i.c.v.) of either: (a) BCM-5, (b) BCM-4, (c) saline solution, or (d) morphine	Danish rural breed rabbits: I: single dose, no further details reported C (a): single dose of 25 μg C (b, c, d): not reported Sprague–Dawley rats: I: single dose of 11–22 μg C (a): single dose of 2.5–12.5 μg C (b): single dose of 100 μg C (c): not reported C (d): single dose of 50 μg	Neurological effect*, anaesthetic function measured with respiratory frequency, tidal volume, inspiratory and expiratory time, etc.	Swedish Medical Research Council (Nr. 2464 and 2862) und “Expressens Prenatalforskningsfond”
13	Kim 2000 [50]	UK	9 Friesian cows, weighing approx. 527–579 kg	To examine the effects of abomasal infusion (or i.v.) of a mixture of three BCMs on insulin in dairy cows	I: Oral administration of BCM mixture containing BCM-4, BCM-5, and BCM-7, with the normal diet C: normal diet	I: single dose of 80 mg of BCM mixture C: not reported	Intermediate markers related to diabetes*, incl. plasma glucose concentration, and serum insulin concentration	The Scottish Executive Rural Affairs Department, The British Council und Korean Collaboration Centre for Biotechnology and Biological Science
14	Lin 1997 [35]	USA	180 male Sprague–Dawley rats, aged 7 weeks	To study the effects of BCM 1–7, 1–5 and 1–4 on food intake of rats adapted to either a high fat (HF) or high carbohydrate (HC) diet; and to compare it to the effects of enterostatin	I: BCM-7 administration (i.p., or i.c.v.) C: Administration (i.p., or i.c.v.) of either: (a) BCM mixture (BCM-1–5), (b) BCM mixture (BCM-1–4), (c) saline solution, or (d) saline solution with naloxone	Various experiments were conducted in which animals received either I or C in different doses	Neurological effect*: behavioural change in food intake (behaviour towards the intake of a high-fat diet and a high-carbohydrate diet)	National Institutes of Health (NIDDK Nr. 45278)
15	Maklakova 1993 [36]	Russia	639 male albino rats (nonpedigree), weighing between 150 and 250 g	To investigate the effects of BCM7 and its des-Tyr-analogues on locomotor activity and the exploratory reaction under different experimental conditions	I: BCM-7 administration (i.p.) C: Administration (i.p.) of either: (a) BCM-4, (b) BCM-6, or (c) distilled water	Single dose of either I or C with the following concentrations: 1 mg/kg, 5 mg/kg, or 20 mg/kg	Neurological effect*: behavioural change (locomotor activity)	Not reported
16	Maslennikova 2008 [37]	Russia	42 new-born outbred albino rats, aged 2–4 days	To evaluate the effect of BCM7 on DNA synthesis in cell populations (tissue) of new-born albino rats	I: BCM-7 administration (i.p.) C: Administration (i.p.) of saline solution	Single dose of either I or C with the following concentrations: 1 mg/kg, or 5 mg/kg	Proliferative process*, measured with DNA Synthesis	Not reported
17	Nedvidkova 1985 [38]	Czech Republic	99 male Wistar rats, weighing 180–200 g	To determine the effect of parenterally given BCM7 and its analogue Tyr-pro-Gly-Pro-Phe Pro-Ile on plasma prolactin release in rats	I: BCM-7 (which has the amino acid sequence: Tyr-Pro-Phe-Pro-Gly-Pro-Ile) administration (i.p.) C: Administration (i.p.) of either: (a) Tyr-Pro-Gly-Pro-Phe-Pro-Ile analogue, (b) Try-Pro-Gly-Pro-Phe-Pro-Ile analogue and naloxone, (c) morphine, (d) morphine and naloxone, or (e) saline solution 0.9%	I: single dose of 15 mg/kg C (a): single dose of 15 mg/kg C (b): single dose of 15 mg/kg and 1.5 mg/kg C (c): single dose of 10 mg/kg C (d): single dose of 10 mg/kg and 1.5 mg/kg C (e): no further details reported	Endocrine function*: serum prolactin concentration	Not reported
18	Panksepp 1984 [20]	USA	Approx. 16 Cornish Rock Broilers, aged between 3 and 5 days	To investigate the effect of different casomorphins on separation induced distress vocalization in young domestic chicks	I: BCM-7 administration (i.c.v.) C: Administration (i.c.v.) of either: (a) BCM-4, (b) BCM-5, or (c) distilled water with acetic acid	Single dose of 50 nmol of either I or C	Neurological effect*: analgesic effect	Not reported
19	Park 2004 [21]	USA	Male Osborne-Mendel rats, aged 10 weeks (no further details reported)	To study the relationship between binding activity and feeding behaviour, we examined the ability of a number of enterostatin analogues (one of them is BCM7) to affect BCM1–7 binding to the F1-ATPase beta -subunit	I: BCM-7 administration (i.c.v.) C: Administration (i.c.v.) of either: (a) enterostatin analogue, or (b) saline solution	I: 1 nmol dissolved in 1 μl saline solution (no further details reported) C (a): 1 nmol dissolved in 1 μl saline solution C (b): 1 μl (no further details reported)	Neurological effect*: behavioural change in food intake (behaviour towards intake of high fat diet)	National Institutes of Health (NIDDK Nr. 45278)
20	Schusdziarra 1983a [44]	Germany	12 dogs, weighing between 25 and 35 kg	To investigate post-prandial insulin release in response to test meals containing opiate-like substances	I: Oral administration of BCM mixture containing BCM-4, BCM-4-amide, BCM-5, and BCM-7, with the normal diet (with and without naloxone) C: saline solution with normal diet	I: 3 mg BCM-7, 3 mg BCM-5, 4 mg BCM-4 and 4 mg BCM-4-amide. If naloxone was administered: 10 mg C: no further details reported	Intermediate marker related to diabetes*: post-prandial insulin release	*Deutsche Forschungsgemeinschaft (SFB 87 G6)*
21	Schusdziarra 1983b [52]	Germany	16 beagle dogs, weighing between 12 and 17 kg	To determine if i.v. infused BCMs affect insulin release in dogs	I: BCM-7 administration (i.v.) C: Administration (i.v.) of either: (a) BCM-3, (b) BCM-4, (c) BCM-5, or (d) Saline solution with albumin	I: 1 nmol/kg/h and 30 min later 100 nmol/kg/h C (a, b, c): 1 nmol/kg/h and 30 min later 100 nmol/kg/h C (d): no further details reported	Intermediate marker related to diabetes*: plasma insulin concentration	*Deutsche Forschungsgemeinschaft (SFB 87 G6)*
22	Schusdziarra 1983c [25]	Germany	8 foxhound dogs, weighing between 24 and 36 kg	To determine the effect of BCMs on postprandial somatostatin release in dogs	I: Oral administration of BCM mixture containing BCM-4, BCM-4-amide, BCM-5, and BCM-7, with the normal diet C: saline solution with normal diet	I: single dose of 12 mg of BCM mixture, which contained 3 mg BCM-7, 3 mg BCM-5, 3 mg BCM-4 and 3 mg BCM-4-amide C: equivalent to the same amount of the intervention	Endocrine function*: post-prandial somatostatin release	*Deutsche Forschungsgemeinschaft (SFB 87 G6)*
23			6 beagle dogs, weighing between 12 and 15 kg		I: BCM-7 administration (i.v.) C: Administration (i.v.) of either: (a) BCM-4, (b) BCM-4-amide, (c) BCM-5, (d) met-encephalin, or (e) saline solution with albumin	1 nmol/kg/h during 30 min either I or C (a, b, c, d, e)	Endocrine function*: post-prandial somatostatin release	
24	Schusdziarra 1983d [26]	Germany	6 beagle dogs, weighing between 12 and 15 kg	To determine the effect of BCMs on postprandial somatostatin release in dogs	I: BCM-7 administration (i.v.) C: Administration (i.v.) of either: (a) BCM-4, (b) BCM-5, (c) saline solution with albumin, (d) morphinhydrochlorid, or (e) Leu-encephalin	Stepwise increasing infusion rate of 1.5 and 100 nmol/kg/h, for 30 min of either I or C (a, b, c, d, e)	Endocrine function*: somatostatin release	*Deutsche Forschungsgemeinschaft (SFB 87 G6)*
25	Sun 1999a [39]	USA	65 male Sprague–Dawley rats, weighing between 250 and 300 g	To find whether BCM7 has any behavioural or analgesic effect in rats	I: BCM-7 administration (i.p.) with and without naloxone C: Administration (i.p.) of saline solution (0.9%)	I: single dose of either 30 ug/kg, 60 ug/kg, or 120 ug/kg. If naloxone was administered: 2 mg/kg C: no further details reported	Neurological effect*: behavioural change and analgesic effect	Robert and Mary Cade Foundation
26	Sun 1999b [40]	USA	35 Sprague–Dawley (Harlan) rats, aged 2–3 months, weighing between 250 and 300 g	To investigate whether BCM7 can cross the blood–brain barrier, to determine which brain areas are affected by BCM7, or to test whether the effect of BCM7 is mediated by opioid receptors	I: BCM-7 administration (i.v.) with and without naloxone C: Administration (i.v.) of saline solution (0.15 M)	I: single dose of either 2.5 μg/kg, 5 μg/kg, 10 μg/kg, or 30 μg/kg. If naloxone was administered: 2 mg/kg C: single dose of 0.5 ml	Neurological effect*: neurologic function (impact on diverse brain regions)	Robert and Mary Cade Foundation
27	Taira 1990 [41]	Finland	45 male Wistar rats, aged 7 days	To study the effects of BCM7 on neonatal sleep in rats	I: BCM-7 administration (i.p.) with and without naloxone C: Administration (i.p.) of saline solution (0.9%)	I: single dose of either 1 mg/kg, 5 mg/kg, 10 mg/kg, 50 mg/kg, or 100 mg/kg. If naloxone was administered: 1 mg/kg C: no further details reported	Neurological effect*: behavioural change (sleeping behaviour)	Not reported
28	Wei 1980 [22]	USA	Male Sprague–Dawley rats, weighing between 200 and 400 g (no further details reported)	To investigate the vagal bradycardia (fall in heart rate) as an index for the bioassay of the in vivo activities of peptides related to enkephalins and to BCMs	I: BCM-7 administration (i.v.) C: Administration (i.v.) of various peptides (no further details reported)	0.05 ml/100 g of either I or C (no further details reported)	Intermediate marker related to CVD *: heart rate	US Public Health grant (USPH Grant DA-00091)
29	White 2000 [42]	USA	19 male Sprague–Dawley rats, aged 10 weeks, weighing between 215 and 240 g	To compare the feeding response to enterostatin and BCM7 injected intragastrically	I: BCM-7 administration (i.g.) C: Administration (i.g.) of sterile water	I: single dose of either 10 nmol, 100 nmol, or 1000 nmol C: not reported	Neurological effect*: behavioural change in food intake (behaviour towards intake of high fat diet)	National Institutes of Health (DK 45278 and DK 32089)
30	Yin 2010 [45]	China	16 male Sprague–Dawley rats, weighing 200–250 g	To investigate the possible effects of BCM-7 against hyperglycaemia and free radical-mediated oxidative stress in streptozotocin-induced diabetic rats	I: Oral administration of BCM-7, with the normal diet C: normal diet	I: daily 7.5 × 10^–8^ mol for 15 days C: normal diet for 15 days	Intermediate marker related to diabetes*, incl. blood glucose- and insulin concentration Food intake Weight	Not reported
31	Yin 2012 [46]	China	16 male Sprague–Dawley rats, weighing 200–250 g	To investigate the protective effects of BCM7 against oxidative stress in pancreas of streptozotocin-induced diabetic rats	I: Oral administration of BCM-7, with the normal diet C: normal diet	I: daily 7.5 × 10^–8^ mol for 15 days C: normal diet for 15 days	Intermediate marker related to diabetes*: oxidative stress in pancreas	China National Science Foundation (Nr. 30871838 and Nr. 30872119)
32	Yin 2019 [27]	China	40 elderly male MK mice, 11 months old; and 10 young KM mice, 2 months old	To investigate whether management with BCM-7 has any effects of regulating intestinal mucosal immunity in aged mice and its possible mechanism	I: BCM-7 administration (i.g.) with normal diet C: saline solution with normal diet	I: daily either 2 × 10^–7^ mol, 1 × 10^–6^ mol, or 5 × 10^–6^ mol for 30 days C: saline solution for 30 days	Gut immune response*, incl. histological analysis of intestinal mucosa, inflammatory cytokines, antioxidant enzymes (SOD, MDA, CAT)	The Natural Science Foundation of the Jiangsu Higher Education Institutions of China (Nr. 16KJB330011 and 17KJB190001), Science and technology innovation fostering fund of Yangzhou University (Nr. 2016CXJ107), and Post-graduates scientific research and innovation projects (Nr. XKYCX18-133)
33	Zhang 2012 [47]	China	16 male Sprague–Dawley rats, weighing about 200 g	To investigate the putative protective effect of BCM7 on diabetic nephropathy in a rat model and to explore the possible mechanisms of this effect	I: Oral administration of BCM-7 C: distilled water	I: daily 7.5 × 10^–6^ mol for 30 days C: not reported	Intermediate marker related to diabetes*: diabetic nephropathy	National Natural Science Foundation of China und Priority Academic Program Development of Jiangsu Higher Education Institutions (PAPD)
34	Zhang 2013 [51]	China	24 male Sprague–Dawley rats, weighing about 200 g	To investigate the effect of BCM7 on the oxidative stress occurring in kidney tissue in streptozotocin-induced diabetic rats and proximal tubular epithelial cells exposed to high glucose	I: Oral administration of BCM-7 C: distilled water	Daily 7.5 × 10^–6^ mol/kg for 30 days either I or C	Intermediate marker related to diabetes*: diabetic nephropathy measured with renal oxidative stress Various blood parameters, incl. enzyme concentrations, glucose, insulin, etc. Weight	National Natural Science Foundation of China, Priority Academic Program Development of Jiangsu Higher Education Institutions (PAPD) und Graduate Students Innovative projects of Jiangsu Higher Education Institutions
35	Zhang 2019 [28]	China	48 male Sprague–Dawley rats, weighing about 250 g (7–8 weeks old)	To investigate the protective effect of BCM-7 and its possible mechanisms on acute kidney injury	I: BCM-7 administration (i.p.) in a septic rat model of acute kidney injury (established by cecal ligation and puncture) C: physiological saline in two control groups of rats: (a): rats without acute kidney injury, or (b): septic rat model of acute kidney injury	I: single dose of 7.5 × 10^–8^ mol C: equal volume as intervention	Kidney index* Kidney damage, incl. histological evaluation, oxidative stress Various blood and urine parameters, incl. creatinine, urea, etc.)	No financial support received
36	Zong 2007 [48]	China	24 female Sprague–Dawley rats, weighing 200–240 g	To investigate the in vivo effect of BCM7 on the regulation of gastric somatostatin and gastrin messenger RNA in rat gastric mucosa	I: oral administration (intubation) of BCM-7, with the normal diet C: oral administration (intubation) of either: (a) saline solution, or (b) poly-Gly-7	I: daily 7.5 × 10^–7^ mol for 30 days C (a): not reported C (b): daily 7.5 × 10^–6^ mol for 30 days	Endocrine function*: gastrin and somatostatin (mRNA expression)	National Natural Science Foundation of China (No. 39770540)

#### Characteristics of studies comparing A1- vs. A2 beta-casein

From the six studies evaluating A1- vs. A2 beta-casein, most studies were performed monocentric in Australia, Poland, India, UK; and one study was carried out as multicentric in New Zealand, Canada, and the UK [12]. At least 723 animals were investigated in all studies together (min 6 animals and max 585 animals per study; one study did not report the number of animals included [13]). The animal population consisted of rats (*n* = 3397), mice (*n* = 339^1^), pigs (*n* = 6), and rabbits (*n* = 60). Animals received the intervention or control intervention orally, mainly supplemented with their normal diet. One study administered the intervention through intubation [15]. The intervention was either A1 milk or A1 beta-casein, whereas the control intervention was A2 milk or A2 beta-casein respectively. The analysed outcomes included gastrointestinal markers and various intermediate markers of diabetes and CVD, including blood glucose and insulin concentrations, blood lipids, and markers of atherosclerosis (i.e., aortic fatty streak and advanced lesions in carotid arteries). Outcomes were measured in living or deceased animals depending on the nature of the outcome (i.e., lipid profile was measured in living animals, bowel inflammation status after bowel resection was measured in deceased animals).

Three studies were financially supported exclusively by independent funding agencies or research institutes [12, 14, 15], two studies reported the a2 Milk Company Limited (formerly A2 Corporation) as sponsor [13, 16], and one study provided no information on funding [17].

All study characteristics can be found in Table 1.

#### Characteristics of studies comparing BCM-7 vs. any other intervention(s)

Thirty-six studies comparing the effects of BCM-7 with other interventions were included. Study characteristics are listed in Table 2. Briefly, all studies were performed monocentric in various countries worldwide, incl. USA, Germany, Russia, and China. The animal population consisted mainly of rats, but also mice, rabbits, cows, broilers, dogs, and cockroaches were analysed. All studies included at least 3154 animals in total (min 6 animals and max 639 animals per study; five studies did not report the exact number of animals included [18–22]). Most studies administered BCM-7 as injection [18–25, 25–42] and ten studies administered it orally [25, 43–51]. Most studies provided BCM-7 alone, whereas three studies used a mix of BCM-fragments (including BCM-7) as intervention [25, 44, 50]. The control interventions were saline solution, distilled water, various proteins (e.g., exorphins and encephalin), or shorter BCM-fragments (e.g., BCM-3, -4, -5). Included studies had mainly an explorative focus on investigating biological effects of BCM-7 (quantitatively and qualitatively) and had no primary interest in comparing BCM-7 with other interventions. Therefore, outcomes measured across studies varied greatly, e.g., pain relief, behavioural change, intermediate markers of diabetes and neurologic functions, various blood parameters (incl. parameters of immune function, enzyme concentrations), etc.

Most studies (*n* = 25) were supported by different independent funding agencies or research institutes, ten studies provided no information on funding [18, 20, 23, 29, 34, 36–38, 41, 45], and one study reported that no funding was received [28].

### Health-related outcomes

Included studies reported on various types of outcomes and most of them were either intermediate markers of a disease or a condition or were measured to describe a physiologic process.

Outcomes investigated across included studies are displayed in bubble charts and described narratively.

#### Details of interventions and health-related outcomes in studies comparing A1- vs. A2 beta-casein

**Fig. 2:**
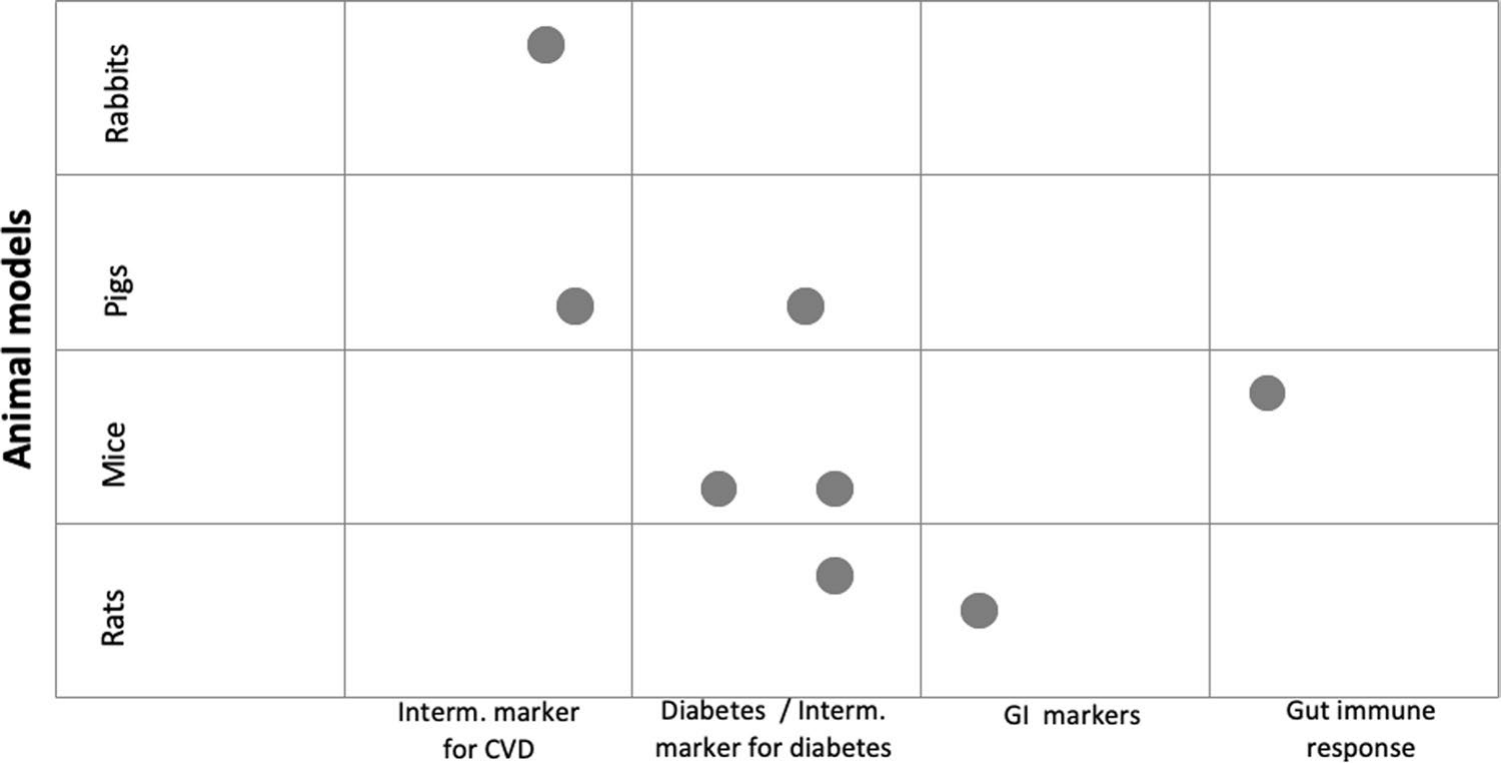
Bubble chart of studies comparing A1- vs. A2 beta-casein. *CVD* cardiovascular, *GI* gastrointestinal. Each dot in the bubble chart represents the primary outcome investigated in the included studies, which are marked with an (*) in Table 1 (i.e., Interm. marker for CVD: blood lipids, aortic fatty streak and lesions in carotid arteries; Diabetes/Interm. marker for diabetes: incidence of diabetes, glucose concentration in blood and urine; GI markers: gastrointestinal transit time and intestinal inflammation; Gut immune response: concentration of immunoglobulins). If a study investigated the primary outcome in more than one animal model, the chart shows a dot for each animal model (i.e., Beales 2002 [12], Kaminski 2012 [15])

Figure 2 shows a bubble chart with the primary outcomes reported in studies comparing A1- with A2 beta-casein. Outcomes are grouped according to the animal model in which they were investigated.

Two studies reported on the following intermediate markers of cardiovascular disease (CVD): blood lipids and aortic fatty streak and lesions in carotid arteries [15, 17]. Tailford et al. reported on significantly fewer aortic fatty streaks formation (as marker of atherogenic effect) in rabbits who were given A2 beta-casein, compared to rabbits under the oral administration of A1 beta-casein [17]. Kaminski et al. found no significant difference in total cholesterol- and triacyglcerols) after A1- or A2 beta-casein administration in pigs [15].

The incidence of diabetes was investigated in four consecutive generations of NOD mice fed with either A1- or A2 beta-casein in the study of Chia et al. [13]. They found that dietary A1 beta-casein increased diabetes incidence in the 3rd and 4th generation of mice, whereas incidence did not change in previous generations [13]. Glucose concentration in blood or urine was investigated in two studies [12, 15]. Authors reported no difference in the glucose concentration in mice and pigs after the administration of A1- or A2 beta-casein [12, 15], whereas a favourable effect of A2 beta-casein compared to A1 beta-casein was observed in the rat population [12].

Furthermore, one study analysed gastrointestinal effects in rats and found that dietary A2 beta-casein administration caused reduced intestinal inflammation and a favourable gastrointestinal transit time compared to the A1 beta-casein intervention [16]. Similarly, Haq et al. found a favourable gut immune response in mice fed A2 beta-casein, compared to mice fed A1 beta-casein (or A1/A2 beta-casein) [14].

None of the included studies investigating A1 vs. A2 beta-casein measured the level of BCM-7, which could provide a link between A1 beta-casein and the release of this bioactive peptide.

#### Details of interventions and health outcomes in studies comparing BCM-7 vs. any other intervention(s)

**Fig. 3:**
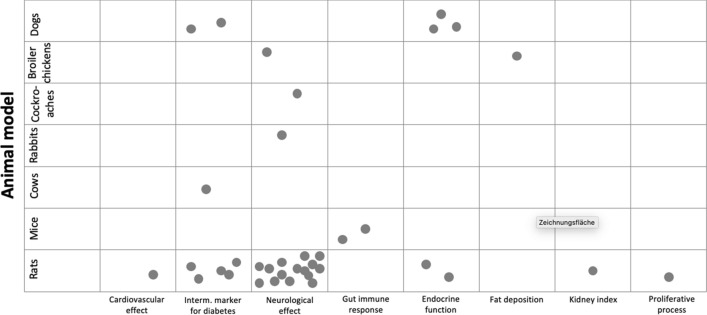
Bubble chart of studies comparing BCM-7 vs. any other intervention. Each dot in the bubble chart represents the primary outcome investigated in the included studies, which are marked with an (*) in Table 2 (i.e., intermediate marker for CVD: heart rate; Interm. marker for diabetes: diabetic cardiomyopathy, glucose and insulin concentration, pancreatic oxidative stress, diabetic nephropathy; Neurological effect: analgesia, behavioural change, anaesthetic function, neurologic function on brain regions; Gut immune response: immunoglobulin concentration, histology of intestinal mucosa; Endocrine function: prolactin concentration, gastrin and somatostatin release). If a study investigated the primary outcome in more than one animal model, the chart shows a dot for each animal model (i.e., Hedner 1987 [19])

Figure 3 shows a bubble chart with the primary outcomes reported in studies comparing BCM-7 with any other intervention(s). Outcomes are grouped according to the animal model in which they were analysed. As mentioned before, studies investigating BCM-7 focused mostly on its biological and metabolic properties such as neurological effects (acting as an opioid). Thus, we herein give an explorative summary about the outcomes that were investigated in included studies, without providing a detailed description of the results of each study.

Briefly, the primary outcomes most analysed in studies investigating BCM-7 were related to neurological effects, such as behavioural change or analgesic effect. The relationship between neurological effects and BCM-7 relies on its ability to cross the blood–brain barrier and act as an opioid peptide. Its interaction with opioid receptors causes exorphin activity in the brain, which unfolds as e.g., behavioural change, analgesic effects, etc. Behavioural change was reported in rats in eight studies and included for example mother-oriented behaviour, learning, sleeping, or food intake behaviour [21, 32, 33, 35, 36, 39, 41, 42]. Most results showed that BCM-7 had indeed a stimulating effect on changing the behaviour of rats receiving this intervention compared to shorter fragments of BCM, saline solution, sterile water, or other equivalent solvent. An additional neurological outcome frequently investigated was analgesic effect, mainly as a pain reliever. Most studies compared this effect to other BCM fragments and investigated the opioid pathway for achieving the analgesic effect, including binding properties (e.g., affinity to opioid receptors). It was analysed in rats in five studies [18, 23, 29–31], in cockroaches in one study [34] and in broiler chickens in one further study [20]. Results showed that BCM-7 achieved significant pain relief in animals receiving the intervention. Many of the mentioned studies investigated additionally the effect of injecting naloxone as a specific opiate-receptor antagonist. They found that the BCM-7 effect was abolished by naloxone, confirming the opiate activity of BCM-7. One additional study reported that rats administered with BCM-7 had a stronger response in various brain regions, compared to rats in the control group [40]. At last, we identified one study reporting on BCM-7 as an anaesthetizing agent in rabbits and found that the intervention decreased respiratory frequency and depressed the central respiratory system, suggesting a positive effect to induce anaesthesia [19].

Eight studies reported on intermediate markers related to diabetes [43–47, 50–52]. From these, two studies found a stimulating effect on post-prandial insulin release in dogs after the intervention with either BCM-7 or a mixture of BCM fragments (incl. BCM-7), compared to shorter BCM fragments or saline solution [44, 52]. Similarly, the study of Yin et al. reported lower blood glucose levels and increased insulin levels in rats fed BCM-7 with their normal diet compared to rats in the control group [45]; and Kim et al. reported that BCM mixture (incl. BCM-7) lowered the responses of serum insulin in cows [50]. Furthermore, three studies found that BCM-7 had a protective effect on diabetic nephropathy in rats [47, 51] and on diabetic cardiomyopathy also in rats [43]; and one study reported less free-radical-mediated oxidative stress in the pancreas of rats fed BCM-7 compared to rats without the intervention [46].

Furthermore, results from five studies were grouped under outcomes related to endocrine function [25, 26, 38, 48]. Specifically, somatostatin release was investigated in three studies in dogs [25, 26], from which two studies found no effect of BCM-7 administration [25], and one study found a dose-dependent inhibition of somatostatin after the administration of BCM-7 [26]. In addition, Zong et al. found that BCM-7 modulated gene expression of the regulatory peptides from G and D cells in a rat population (by means of the paracrine action of somatostatin) [48]. One further study found an increased serum prolactin concentration in the blood of rats after BCM-7 [38], suggesting the beneficial effect of BCM-7 in lactation and in regulating the immune function.

Gut immune response was reported in two studies. Haq et al. found that oral administration of BCM-7 increased gut immune response in mice (measured with immunoglobulins and the concentration of various enzymes) [49], and similarly Yin et al. reported an improvement of intestinal mucosal immune decline (induced by aging) in mice administered BCM-7 [27].

Finally, four studies reported on further different outcomes. Chang et al. found increased growth performance with the administration of BCM-7 and described the involved mechanisms in broiler chickens [24]. Similarly, one study showed that BCM-7 stimulated DNA synthesis in new-born rats [37]. Furthermore, Wei et al. found no effect of BCM-7 on the heart rate of rats (acting as opioid agent) [22]. Finally, Zhang et al. found that BCM-7 was able to reduce sepsis in rats with induced kidney injury [28].

## Discussion

### Principal findings

The aim of this scoping review was to identify all potential health outcomes associated with A1 beta-casein (or BCM-7) in animal studies, to define the number and types of available animal studies, and to identify any gaps in the evidence base. To our knowledge, this is the first systematic scoping review on this topic in animal studies. We included and synthesized 42 eligible studies, most of them had an explorative character to investigate physiological properties of BCM-7 as an opioid peptide, for example on neurological functions. In this sense, BCM-7 was described to act as analgesic agent and to influence various metabolic processes. Only six included studies evaluated the effect of A1 milk (or A1 beta-casein) compared to A2 milk (or A2 beta-casein) and results suggest that A2 milk could have beneficial gastrointestinal effects compared to A1 milk. However, results for other outcomes e.g., outcomes related to CVD and diabetes seem inconclusive. We recently published a systematic review about the health effects of A1- and A2 beta-casein in humans and the results regarding gastrointestinal effects pointed in the same direction as results found in this scoping review in animal studies [5] and as the results from the systematic review of Brook-Taylor et al. [4]. Although effects from animal studies cannot be comparable to effects in humans, results from animal studies, together with further epidemiological and experimental studies (incl. in-vitro, biochemistry, pharmacological studies), could complement research for humans, particularly when the body of evidence remains uncertain as is the case in the A1- and A2 milk debate [4, 5]. For example, neurological effects of A1 beta-casein (or BCM-7) on schizophrenia, autism, or ADHS, where evidence is very limited and studies in humans are difficult to perform, could be expanded with results from animal research.

We identified 14 references which were not included in the scoping review due to the reasons mentioned above but results from these studies could influence the evidence map presented here. Additionally, effects of the consumption of A2 milk are increasingly being investigated parallel to the fast commercialization of A2 milk all over the world. Therefore, it will remain as an emergent topic for the next years and an update of this scoping review, or even a full systematic review would certainly be fundamental in the near future.

### Strengths and weaknesses of the scoping review

To our knowledge, this is the first scoping review summarizing all available evidence on A1- and A2 beta-casein in animal studies. The summary of our results provides an overview of research performed so far in this field. Included studies were extremely heterogeneous in terms of the study population (e.g., healthy animals, but also animals with certain induced health issues were included), type of intervention and dose, and type of outcome measures; therefore, one of the main challenges during this scoping review was to synthesize available evidence in a comprehensive manner. Many of the included studies investigating BCM-7 as the intervention had as primary objective to explore biological effects and possible mechanisms of this opioid peptide. Thus, a clear health-related outcome was hardly identified. We are presenting the results separated by type of comparison (A1- vs. A2 milk; and BCM-7 vs. any other intervention) to provide results as clear and transparent as possible. Some important data to be considered when performing systematic reviews of animal studies were extracted but not presented in this scoping review (e.g., housing conditions, acclimatisation, etc.) to maintain a sound overview of this topic. For example, data about blinding of investigator or outcome assessor was extracted but no included study reported on this. To drive conclusions about this, further investigation is needed (e.g., contacting study authors to confirm if blinding was performed and how), but was not planned during this scoping review. Considering that this emerging topic is being actively investigated, not only in human studies but also in animal models, it is possible that new results relevant to this scoping review will emerge in the near future.

It is important to mention that the present work describes the scope of this topic without further critical evaluation of the included primary studies, e.g., no risk of bias assessment was performed. Therefore, conclusions about the effects of the intervention on health-related outcomes were not driven. Performing a systematic review would certainly provide a deeper insight into the evidence base on the health effects of A1- and A2 beta-casein.

## Conclusion

Most studies investigated physiological properties of BCM-7 and only six studies compared the effects between A1- and A2 beta-casein. More studies on animal models would provide essential information to inform research gaps and results from these studies could eventually complement research for humans, particularly when the body of evidence remains uncertain as is the case in the A1- and A2 milk debate.

## Supplementary Information

Below is the link to the electronic supplementary material.

Supplementary file1 (DOCX 25 KB)

Supplementary file2 (DOCX 14 KB)

Supplementary file3 (DOCX 14 KB)

## Data Availability

The datasets supporting the conclusions of this article are included within the article (and its additional files 1, 2, and 3).

## Footnotes

1 This is underestimated, since two studies provided no information on the number of animals included.
