# Supplementary material for: A1- and A2 beta-casein on health-related outcomes: a scoping review of animal studies
Source: Eur J Nutr. 2021 Jun 1;61(1):1–21. doi: 10.1007/s00394-021-02551-x (PMC8783860; doi:10.1007/s00394-021-02551-x)
Supplement: Supplementary file 3 — Supplementary file3 (DOCX 14 KB) [file 394_2021_2551_MOESM3_ESM.docx]

|  | **Author** | **Title** | **Year of publication** | **Reason for exclusion** |
| --- | --- | --- | --- | --- |
| 1 | Brantl et al. | Phamacological properties of β-casomorphins | 1980 | Published only as abstract |
| 2 | Dubynin et al. | Delayed neurotropic effects of beta-casomorphin 7 chronically administered to newborn albino rats | 1999 | Full-text in other language than English, German, Spanish, or French |
| 3 | Dubynin et al. | Changes in beta-casomorphine-7 effect on behavior of albino rat pups in postnatal development | 2001 | Full-text in other language than English, German, Spanish, or French |
| 4 | Dubynin et al. | Behavioural effects of beta-casomorphin-7 in its intranasal administration | 2004 | Full-text in other language than English, German, Spanish, or French |
| 5 | Dubynin et al. | Influence of acute and chronic administrations of beta-casomorphins on the maternal motivation in albino rats | 2005 | Full-text in other language than English, German, Spanish, or French |
| 6 | Fagbodun et al. | Effect of A1 versus a2™ milk exposure at an early developmental age on the endogenous opioid system of the rat brain | 2019 | Published only as abstract |
| 7 | Maklakova et al. | Effect of beta-casomorphine-7 on different types of training of white rats | 1995 | Full-text in other language than English, German, Spanish, or French |
| 8 | Maklakova et al. | The effect of beta-casomorphin-7 on the level of food and defense motivations in different types of learning | 1995 | Full-text in other language than English, German, Spanish, or French |
| 9 | Maklakova et al. | Sex-dependent differences in the action of chronically administered beta-casomorphin-7 on the behavior of white rat pups | 1996 | Full-text in other language than English, German, Spanish, or French |
| 10 | Steinerová et al. | Does artificial suckling nutrition pose a risk of atherosclerosis at the adult age? | 2006 | Full-text in other language than English, German, Spanish, or French |
| 11 | Trivedi et al. | Preclinical and clinical trials for investigating the effect of A1 vs A2 beta-casein containing diet on glutathione antioxidant status | 2016 | Published only as abstract |
| 12 | Trivedi et al. | Effect of A1 vs A2 beta-casein containing diet on glutathione antioxidant status: Implications for inflammation and cognitive function via gut-brain axis | 2017 | Published only as abstract |
| 13 | Wang et al. | Protective effect and mechanism of beta-CM7 on renin angiotensin system & diabetic cardiomyopathy | 2016 | Full-text in other language than English, German, Spanish, or French |
| 14 | Zozulia et al. | Naloxone-induced suppression of the behavioral manifestation of serotoninergic system hyperactivation by beta-casomorphins-7 in mice | 2009 | Full-text in other language than English, German, Spanish, or French |
